# Adipose tissue-derived extracellular fraction characterization: biological and clinical considerations in regenerative medicine

**DOI:** 10.1186/s13287-018-0956-4

**Published:** 2018-08-09

**Authors:** Barbara Bellei, Emilia Migliano, Marinella Tedesco, Silvia Caputo, Federica Papaccio, Gianluca Lopez, Mauro Picardo

**Affiliations:** 1 Laboratory of Cutaneous Physiopathology and Integrated Center of Metabolomics Research, San Gallicano Dermatologic Institute, IRCCS, Via Elio Chianesi, 53 Rome, Italy; 2 Department of Plastic and Reconstructive Surgery, San Gallicano Dermatologic Institute, IRCCS, Rome, Italy

**Keywords:** Growth factors, Adipose tissue, Stem cells, Regenerative medicine, Skin

## Abstract

**Background:**

Adipose tissue-derived stem cells are considered to be a promising source in the field of cell therapy and regenerative medicine. In addition to direct cell replacement using adipose tissue or purified stem cells, intercellular molecule exchange by the adipose tissue complex, a vast array of bioactive secretory factors, demonstrated beneficial effects by reducing tissue damage and stimulation of endogenous repair. However, for therapeutic purposes, the use of secretome derivatives, such as full conditioned media or purified exosomes generated in vitro, may present considerable disadvantages for cell manufacturing, storage, product safety, and their potential as a ready-to-go therapeutic product.

**Methods:**

In this study, the effect of a liquid fraction of lipoaspirates isolated intraoperatively from 28 healthy donors was evaluated for their protective effect against oxidative stress and senescence, proliferation, and migration in vitro on normal human melanocytes, keratinocytes, and fibroblasts. Immunoenzymatic quantification of several growth factors and important signal molecules was used to define the biological profile of physiological adipose tissue secretome.

**Results:**

Adipose tissue extracellular fraction (AT-Ex), isolated from lipoaspirate, exhibited significant potential for skin repair. AT-Ex augmented dermal and epidermal cell proliferation in a dose-dependent manner without promoting cancer cell growth. Moreover, migration of dermal fibroblasts, an important phenomenon implicated in endogenous repair, was enhanced by AT-Ex treatment. AT-Ex has a positive impact on oxidative stress damage when cells are exposed to extrinsic hostile factors and prevent a fibroblast senescence phenotype including paracrine functions associated with skin aging.

**Conclusions:**

Collectively, our findings propose natural systems carrying the physiological balance of in-vivo produced secretome that could improve cutaneous wound healing and tissue repair. This approach, representing an innovative perspective and therapeutic strategy in regenerative medicine, could also be combined with autologous stem cell grafts to treat chronic nonhealing wounds, stable vitiligo, severe burns, and post-oncological scarring.

**Electronic supplementary material:**

The online version of this article (10.1186/s13287-018-0956-4) contains supplementary material, which is available to authorized users.

## Background

Over the past decade, adipose tissue has gained significant importance in tissue engineering/regenerative medicine fields due to recent biotechnological advances and the redefinition of the classic “autologous tissue transplant” into more customizable treatment options useful in a variety of different applications in plastic surgery. Adipose tissue is a multifunctional organ that contains various cellular types, such as mature adipocytes and the stromal vascular fraction (SVF), a source of adipose-derived stem cells (ADSCs), endothelial progenitor cells, pre-adipocytes, lymphocytes, mast cells, pericytes, and adipose-resident macrophages with repair and regenerative potential [1–3]. ADSCs represent a viable alternative to bone marrow-derived mesenchymal stem cells due to the demonstration of higher mesenchymal stem cell concentration [4, 5] and ease and safety of access in the native adipose tissue. The dominant therapeutic mechanism by which a fat graft participates in tissue repair has been explained by cell replacement due to the multilineage differentiation capacity of mesenchymal stem cells [6–8]. Recent discoveries suggest that the adipose-derived extracellular fraction, containing a multitude of bioactive peptides including growth factors and cytokines, possesses similar protective and reparative properties as their cellular counterparts in tissue repair [9–14]. Multiple recent studies have documented the composition of adipose tissue secretome [15–18], also comparing adipocyte secretome with the SVF secretome [19]. However, due to a lack of uniformity in the methodological approach, the clinical relevance of this adipose tissue-associated component needs a definitive conclusion. It is also necessary to take into consideration that the secretory profile of SVF cells, adipocytes, or adherent adipose-derived mesenchymal stem cells cultured in vitro, alone or in combination as a mixed population, will never be truly compliant with the in-vivo conditions where the multiple cell types that comprise adipose tissue are in close proximity to each other. Consequently, as part of normal tissue function, there is significant cross-talk between the cells which occurs through direct cell-cell contact or surface molecular receptors and secreted bioactive factors. Additionally, matrix components of adipose tissue represent a unique physical environment essential for the maintenance of the stem cell niche. The published studies mostly report data collected in in-vitro cultures that are generally maintained in serum-containing media before sample collection. To avoid exogenous serum contaminants, some researchers use, wash, and maintain cells in a serum-free medium, which is a stressful situation for the cells [20]. Certain groups have analyzed the behavior of the ADSC secretome in the context of a proinflammatory stimulus [21]. In order to gain a more comprehensive and useful understanding of this biological system, especially for predicting the clinical effects of the adipose tissue extracellular components, ideally studies should be based on a more realistic model, as much as possible without in-vitro manipulation. Moreover, according to the criteria indicated by the regulatory authorities, the material usable for therapeutic purposes should be minimally manipulated thus excluding the possibility of using lipoaspirate derivates generated in vitro. Accordingly, in this study we analyzed the biological activity of adipose tissue lipoaspirate extracellular fraction (AT-Ex), as obtained during standard surgical procedures, on the major skin cell types for regenerative purposes. We found that whole adipose tissue secretome is able to stimulate epidermal and dermal cell proliferation in a dose-dependent manner, delay apoptosis, and enhance dermal fibroblast migration. Moreover, bioactive ingredients of the AT-Ex secretome help to overcome stress-induced premature senescence of fibroblasts. Furthermore, AT-Ex enhances the proliferation rate of adipose-derived mesenchymal stem cells, increasing the therapeutic potential of autologous grafts.

Considering all these data, we propose the use of biological activities of the lipoaspirate liquid phase as an innovative cell-free therapy to stimulate endogenous repair for specific applications requiring functional or structural tissue regeneration but not soft tissue augmentation, such as nonhealing wounds, severe burns, vitiligo, hypertrophic scarring, and esthetic medicine.

## Methods

### Ethic statement

The Declaration of Helsinki Principles were followed and patients gave written informed consent to collect samples of human material for research. Furthermore, the Institutional Research Ethics Committee (Istituti Regina Elena e San Gallicano) approved all research activities involving human subjects.

### Sample collection

#### Surgical procedure and adipose tissue sampling

All samples were waste materials collected as a by-product of esthetic surgery. Fat tissue was harvested under general anesthesia from the abdominal region with a 3-mm blunt cannula by standard sterile liposuction techniques as described by Coleman and Katzel [22] with infiltration of Kleine’s solution (30 cm^3^/100 cm^2^) using 20-cm^3^ Luer-lock syringes. Adipose tissue was collected during surgery, immediately transported to the laboratory, and processed on receipt.

##### AT-Ex fraction preparation

To separate the liquid phase, the harvested material collected in syringes (~ 8 mL) was processed using two different methods: 1) centrifugation according to the technique of Coleman and Katzel (3 min at 3000 rpm, 1811 *g*) and then left to decant for an additional 10 min; and 2) a sedimentation step (20 min). In both cases, the recovered liquid phase (lower phase) was centrifuged at 300 *g* for 5 min to remove debris and then filtered through a 0.22-μm filter system (Millipore Merck, Milan, Italy). The study was performed using AT-Ex from 28 different healthy donors (19 females and 9 males) with an age range of 19 to 66 years. The upper phase containing the fat tissue was further treated to isolate adipose stem cells as discussed below.

##### ADSC isolation

ADSCs were obtained by adipose tissue (2–3 mL) digestion with collagenase A (Sigma Aldrich, Milan, Italy) as previously reported [23] and then seeded onto a T25 flask at 37 °C, 5% CO_2_ in Dulbecco’s modified Eagle’s medium (DMEM) containing 10% fetal bovine serum (FBS) and antibiotics to select adherent cells. ADSC cell lines (*n* = 5) at passages 3–6 were used for the experiments.

#### Cell cultures

Dermal and epidermal cells were isolated from healthy individuals (20 females and 18 males, age range 19 to 79 years, average 48.6 years) who had undergone plastic surgery. Primary cultures of normal human keratinocytes (NHK; number of cultures = 16) were maintained in M154 medium with Human Keratinocytes Growth Supplement (HKGS; Cascade Biologics Inc., Portland, OR USA) plus Ca^2+^ (0.07 mM), and antibiotics. Normal human melanocytes (NHM; number of cultures = 16) were maintained in M254 medium with Human Melanocytes Growth Supplement (HMGS; Cascade Biologics Inc.,) and antibiotics, and normal human fibroblasts (NHF; number of cultures = 27) were maintained in DMEM containing antibiotics (EuroClone S.p.A., Milan Italy) supplemented with 10% FBS (Hyclone Laboratories, South Logan, UT, USA). All the experiments were performed using cells from short-term cultures (2–10 cell culture passages for NHM and NHF and 1–4 cell culture passages for NHK). A431 and 1300-UC squamous carcinoma cell lines were cultured in DMEM containing 10% FBS whereas Mel501 and M14 melanoma cells were maintained in RPMI containing 10% FBS, both in the presence of antibiotics.

#### Proliferation assays

The effects of AT-Ex and plasma on cell cultures were determined using the MTT colorimetric assay (Sigma Aldrich). Briefly, 2 × 10^4^ keratinocyte and melanocytes, or 0.8 × 10^4^ fibroblasts, were seeded into 24-well plates for 24 h to adhere. The standard growth medium was then changed with medium containing AT-Ex or plasma at the appropriate concentrations (1%, 2%, 5%, and 10% v/v) in the presence or not of serum (NHF, Mel501, and M14) or in the presence or not of specific growth supplements (HMGS or HKGS) and left to grow for 3 and 6 days before being incubated with 3-(4,5 dimethylthiazol)-2,5-diphenyl tetrazolium bromide (MTT) for 2 h. After this time, the medium was removed and the resulting crystals were solubilized in dimethyl sulfoxide (DMSO). The absorbance was measured at 570 nm with a reference wavelength of 690 nm. Absorbance readings were subtracted from the value of blank wells, and the increase in cell growth was calculated as a percentage of absorbance with respect to control samples. Experiments were performed in duplicates. To exclude that AT-Ex acts on cell metabolic activity rather than on cell proliferation, cell counts were performed after 72-h treatment by Trypan blue exclusion assay.

#### Cell culture treatments

The exposure doses were chosen that corresponded to approximately 60% cell viability 24 h after treatment, as measured by the MTT assay as determined in preliminary dose-dependent experiments (data not shown). One hour before cell treatment the culture medium was replaced with starved fresh medium without phenol red and left to equilibrate in a humidified atmosphere of 5% CO_2_ at 37 °C for 1 h; cells were then irradiated with an appropriate dose of ultraviolet (UV). A second group of cells was incubated for 1 h in full-starved medium containing H_2_O_2_ at the selected concentration. The control cells were incubated in parallel without irradiation or hydrogen peroxide treatment. To examine the effect of AT-Ex and plasma on cells, immediately after treatment cells were incubated in fresh medium containing 2% treatments (or not as control cells). Post-treatment H_2_O_2_ experiments were performed in the absence of FBS. Cell survival rates were measured by MTT assay as described above. Experiments were performed in triplicate.

#### Scratch assay

Cells were seeded on 35-mm plates and allowed to grow until confluence. At this time, regular growth medium was removed and, after cell washing, replaced with starved medium (DMEM without serum for fibroblasts and M154 without HKGS for keratinocytes). The cell monolayer was then scratched to create a standardized cell-free area using a 200-μl pipette tip, and cellular debris was washed with phosphate-buffered saline (PBS). Cell culture medium with AT-Ex or plasma (2%) was added and incubated for 18 h. Images were recorded using an Axiovert 25 inverted microscope (Carl Zeiss, Oberkochen, Germany) and a Power Shot G5 digital camera (Canon, Inc., Tokyo, Japan) immediately after the scratch (T0) and at 18 h. The extent of wound closure was presented as the percentage by which the original scratch width had decreased. Quantification of cell migration was performed using the TScratch method.

#### Psoralen and ultraviolet A (PUVA) treatment

8-Methoxypsoralen (8-MOP) (Sigma-Aldrich) was added to the cell culture medium at 25 ng/ml for 4 h. Cells were irradiated at a dose 6 J/cm^2^ in DMEM without phenol red and FBS containing 8-MOP using a Bio-Sun irradiation apparatus (Vilbert Lourmat, Marne-la-Vallee, France) with maximum emission at 365 nm in the UVA spectral region (340–450 nm). Following irradiation, the medium was replaced with fresh medium, which was changed twice a week thereafter. AT-Ex treatment was added at 2% immediately following PUVA and refreshed at each medium change. PUVA-treated control cells were cultured with 2% FBS. Experiments were stopped 2 weeks after PUVA treatment since at this time point senescent biomarkers are stably expressed.

#### Semiquantitative real-time polymerase chain reaction (RT-PCR)

Total RNA was extracted using the Aurum Total mini kit (BioRad, Milan Italy). cDNA was synthesized from 1 μg total RNA using the FirstAid kit (Fermentas, ThermoFisher Scientific, Waltham, MA, USA) and amplified in a reaction mixture containing SsoAdvanced Universal Syber Green Supermix (BioRad) and 25 pmol forward and reverse primers using a CXF96 Touch Cycler (BioRad). All samples were run in triplicate. Amplification of the β-actin (βact) transcript from each sample was included as the internal control. Sequences of primers (intron spanning) can be found in Additional file 1 (Table S1). For each gene, the assessment of quality was performed by examining PCR melt curves after quantitative (q)RT-PCR to ensure product specificity.

#### Western blot analysis

Cell extracts were prepared with RIPA buffer containing proteases and phosphatases inhibitors. Proteins were separated on SDS-polyacrylamide gels, transferred to nitrocellulose membranes, and then treated with the following primary antibodies: anti-Fibronectin mouse monoclonal (1:1000) anti-N-cadherin rabbit polyclonal (1:1000; Santa Cruz Biotechnology Inc., Santa Cruz, CA, USA), anti-Mn-SOD and anti-HO-1 (1:500; Stressgen Biotechnology Corporation, CA, USA), anti-actin α-Smooth Muscle monoclonal (1:1000) and anti-Catalase (1:1000; Sigma Aldrich), anti-VEGF rabbit polyclonal (1:200; Abcam, Inc., Cambridge UK), and anti-PPARγ (1:500; Cell Signaling Technology, MA, USA). Anti-β-tubulin I mouse monoclonal antibody (1:5000; Sigma Aldrich) was used to normalize protein content. Horseradish peroxide-conjugated goat anti-mouse or goat anti-rabbit secondary antibody complexes were detected by chemiluminescence (Cell Signaling Technology). Imaging and densitometry analysis were performed with the UVITEC Mini HD9 acquisition system (Alliance UVItec Ltd., Cambridge, UK).

#### ELISA assay

Growth factor and bioactive molecule concentrations in AT-Ex and plasma were measured using commercially available enzyme-linked immunosorbent assay (ELISA) kits according to the manufacturer’s instructions: basic fibroblast growth factor (bFGF; RayBio, Inc., Norcross, GA, USA), epidermal growth factor (EGF), erythropoietin (Epo), granulocyte/macrophage colony-stimulating factor (GM-CSF), and vascular endothelial growth factor (VEGF)-A (eBioscience, Inc. San Diego, CA, USA), keratinocyte growth factor (KGF; Cohesion Biosciences Ltd., London, UK), wingless type (Wnt)3a and Wnt10b (Cloud-Clone Corp., Katy, TX, USA), hepatocyte growth factor (HGF), stem cell factor (SCF), and α-melanocyte stimulating hormone (α-MSH; Cusabio Technology LLC, Baltimore, MD, USA). Matrix metalloproteinase (MMP)2 (4Abio Co. Ltd., Slough, UK) and Elastin (Elabiosciences, Houston, TX, USA) release in culture medium by photodamaged cells was measured after 2 weeks of treatment and results were normalized against protein concentration.

#### Quantification of catalase and superoxide dismutase (SOD) activity

Catalase and SOD activity were measured using the DetectX Catalase Fluorescent Activity Kit and the DetectX Superoxide Dismutase Colorimetric Activity Kit (Arbor Assay, Tema, Ricerca, Italy) according to the manufacturer’s instructions. Sample were diluted 1:5 prior to assay. Experiments were performed in duplicates.

#### Senescence-associated β-galactosidase (SA-β-gal) staining

After 2 weeks, PUVA-treated cells were fixed and stained as indicated by the Senescence β-Galactosidase Staining kit (Cell Signaling Technology). Stained cells were imaged using an Axiovert 25 inverted microscope (Carl Zeiss, Oberkochen, Germany) and a Power Shot G5 digital camera (Canon Inc., Tokyo, Japan).

#### Immunofluorescence analysis

Cells on coverslips were fixed with 4% paraformaldehyde for 20 min at room temperature followed by 0.1% Triton X-100 to allow cell permeabilization. Cells were then incubated with the following primary antibodies: anti Ki-67 (1:500; Abcam), anti-E-cadherin monoclonal antibody (1:500; Dako Corp., Carpinteria, CA, USA), and anti Cx43 (Sigma Aldrich) for 1 h. Primary antibodies were visualized using anti-rabbit IgG or anti-mouse IgG Alexa Fluor 488 (BD Biosciences, Milan, Italy). Nuclei were visualized with 4′,6′-diamino-2-phenylindole (DAPI). Fluorescence signals were recorded using a CCD camera (Zeiss, Oberkochen, Germany).

#### Flow cytometric analysis: quantification of apoptotic cells

Cell death and apoptosis were analyzed by the annexin-V FITC/propidium iodide (PI) double staining method. Cells were harvested by trypsinization, resuspended in the staining buffer (10 mM HEPES/NaOH, pH 7.4, 140 mM NaCl, 2.5 mM CaCl_2_), stained with FITC-labeled annexin V and PI for 15 min at room temperature in the dark, and then kept on ice until analysis. Production of reactive oxygen species (ROS) was assessed with the fluorescent dye 2’7’-dichlorodihydrofluorescein diacetate (H_2_DCFDA; Sigma Aldrich). Cell permeable, nonfluorescent H_2_DCF is oxidized to the highly fluorescent dye 2’7’-dichlorofluorescein (DCF) in the presence of intracellular ROS. Cells were incubated at 37 °C for 30 min with 2.5 μM H_2_DCF for 30 min at 37 °C and 5% CO_2_ in phenol red-free full-starved medium in the dark. After removing the probe solution, the cells were washed with PBS, trypsinized, centrifuged at 1000 rpm, and then resuspended in PBS. After oxidation of H_2_DCF into fluorescent DCF by ROS, signals were measured by flow cytometry. Cytofluorometric analysis of mitochondrial membrane potential was performed using the J-aggregate forming lipophilic cation 5,5′,6,6′-tetrachloro-1,1′,3,3′-tetraethyl-benzimidazolyl-carbocyanine iodide (JC-1, Sigma Aldrich). Samples were cultured with 5 μg/mL JC-1 staining solution for 20 min at 37 °C, avoiding direct light, and then washed twice with PBS. The fluorescence intensity of mitochondrial JC-1 monomers (λEx 490 nm, λEm 530 nm) and aggregates (λEx 525 nm, λEm 590 nm) were detected immediately. For all flow cytometric analysis, data from 2 × 10^4^ cells were acquired using a FACSCalibur (Becton Dickinson) and analyzed using FlowJo or CellQuest software. The median fluorescence intensity (MFI) analysis was performed in a linear scale. DCFH-DA fluorescence was analyzed on gated viable cells.

#### Statistical analysis

Single experiments and results in the figures are representative of several experiments performed with at least five cell lines from different donors. Quantitative data are reported as mean ± standard deviation (SD). The data were statistically analyzed using Student *t* test. A *p* value of less than 0.05 was considered significant. All original data are available for any revisions.

## Results

### Adipose-derived extracellular fluid promotes cell proliferation

**Fig. 1:**
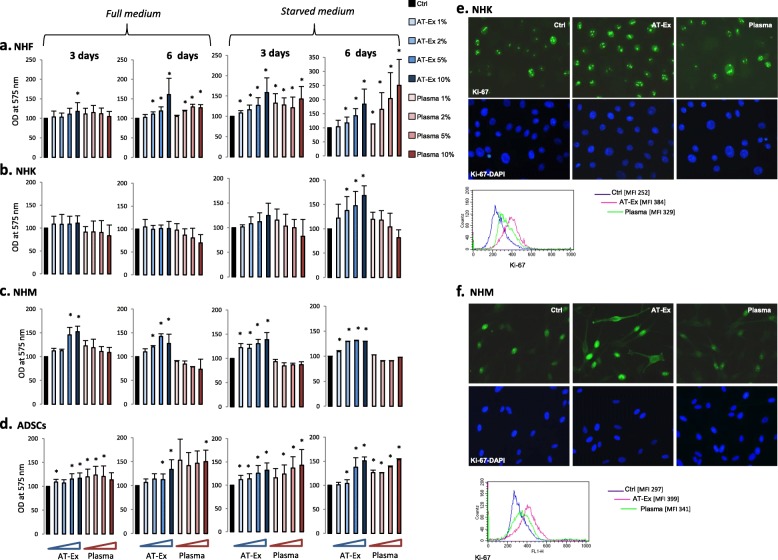
AT-Ex increases the proliferation rate of normal cells. Normal human fibroblasts (NHF) (**a**), normal human keratinocytes (NHK) (**b**), normal human melanocytes (NHM) (**c**) and adipose-derived stem cell (ADSC) (**d**) cell cultures were grown in the presence of increasing doses (1%, 2%, 5%, and 10% v/v) of adipose tissue extracellular fraction (AT-Ex) or matched patient plasma for 3 and 6 days. Experiments were performed in full medium and starved medium. Cell proliferation was measured by MTT assay. Data represent the mean ± SD of 14 (NHK), 12 (NHM), 24 (NHF) and 5 (ADSCs) different experiments performed in duplicate; statistical significance versus untreated control is reported as *p < 0.05. **e** Immunofluorescence analysis of NHK and **f** NHM cells treated with AT-Ex or matched plasma (2%) for 24 h in starved medium was used to evaluate the expression of the proliferation marker Ki-67. Untreated cells were used as a reference. Nuclei were labeled with bisbenzidine (DAPI). Original magnification 40×. Images are representative of several independent experiments. Additionally, for immunofluorescence quantification, median fluorescence intensity (MFI) was measured by FACS analysis. Histogram plots report cell distribution of AT-Ex, plasma, and untreated (Ctrl) cells. OD optical density

**Fig. 2:**
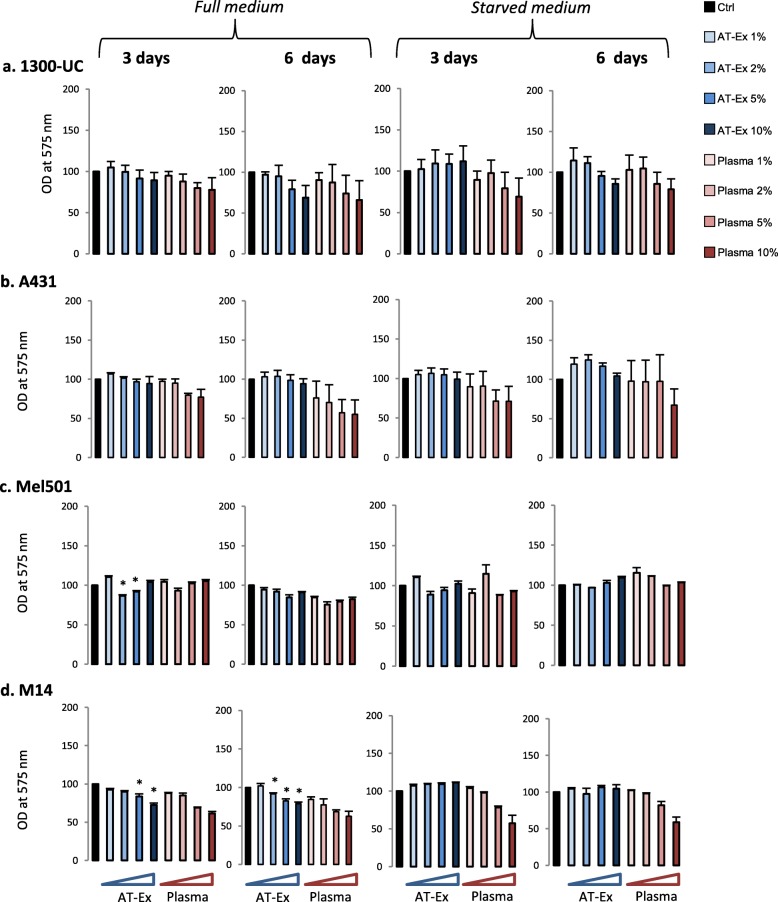
The presence of adipose tissue extracellular fraction (AT-Ex) did not promote cancer cell growth. 1300-UC (**a**) and A431 (**b**) carcinoma cells, and Mel501 (**c**) and M14 (**d**) melanoma cell cultures were grown in the presence of increasing doses of AT-Ex or matched patient plasma for 3 and 6 days. Experiments were performed in full medium and starved medium. Cell proliferation was measured by MTT assay. Data represent the mean ± SD of five different experiments performed in duplicate; statistical significance versus untreated control (Ctrl) is reported as *p ≤ 0.05. OD optical density

To characterize the effects of AT-Ex on dermal and epidermal cell proliferation we examined the responses of normal human keratinocyte (NHK), melanocyte (NHM), and fibroblast (NHF) cell cultures in a set of dose-dependent experiments. The results at days 3 and 6 were chosen since these time points correspond, respectively, with the logarithmic growth phase during which the cells proliferate significantly and to the endpoint of the growth curve. In standard cell culture conditions (full medium containing FBS or HMGS or HKGS), as well as in minimal (starved) medium, AT-Ex-treated cells increased the proliferation rate compared with control untreated cells (Fig. 1a–c). The mitogenic effect was lower for NHK, compared with NHM and NHF, and reached statistical significance only in starved medium. Results were additionally confirmed on ADSC cultures previously isolated from lipoaspirates and maintained in vitro for experimental purposes (Fig. 1d). In contrast, the effect of donor-matched plasma displayed pleiotropic effects depending on the cell type. For NHF, supplementation with plasma results in a similar and in some cases higher stimulation of cell growth with respect to AT-Ex, whereas NHM and NHK slowed cell proliferation. ADSCs, similar to NHF, took advantage of both AT-Ex and plasma supplementation suggesting that ADSC grafts could benefit from extracellular fraction adjuvant therapy. To exclude the impact of AT-Ex on the metabolic activity of cells rather than on cell proliferation, we additionally performed cell counts after 72 h treatment (Additional file 1: Figure S1). Immunostaining for the nuclear proliferation marker Ki-67 correlated with growth curve results and confirmed the absence of a significant mitogenic effect in keratinocyte and melanocyte cultures treated with plasma (Fig. 1e, f). In the case of NHK, phase-contrast microscopy observation revealed the induction of morphological changes compatible with elevation of calcium (Additional file 1: Figure S2A). This hypothesis is consistent with high concentration of calcium in plasma estimated at 2.1–2.8 mmol/L [24, 25]. According to the elevation of extracellular calcium, immunofluorescence analysis revealed the prevalent specific localization of E-cadherin adhesion protein on the plasma membrane (Additional file 1: Figure S1B). The addition of serum and elevation of calcium have been reported to induce premature differentiation of keratinocytes in vitro [26] and, for this reason, a serum-free system is used for studies on growth control in keratinocytes. One of the challenges concerning transplantation of adipose tissue derivate is the possibility of promoting an oncogenic potential of the cells. One of major concerns in the application of regenerative therapies during cancer remission is the possible triggering of cancer recurrence. Considering that one of the possible applicative clinical targets for AT-Ex treatment is post-oncological scarring, we evaluated whether treatment with lipoaspirates was associated with the potential risk of increased proliferation of cancer cells, skin-derived carcinoma, and melanoma cells when treated with AT-Ex samples. Most of the lipoaspirates tested did not modify the proliferation rate of cancer cells (Fig. 2). In general, AT-Ex did not impact on carcinoma cell proliferation. In contrast, on M14 melanoma cells, and in line with previous studies on lung cancer and leukemia cells treated with stem cells secretome [11, 27], AT-Ex exerted a moderate cytostatic effect.

### Lipoaspirate extracellular fraction stimulates cell migration and extracellular tissue remodeling

**Fig. 3:**
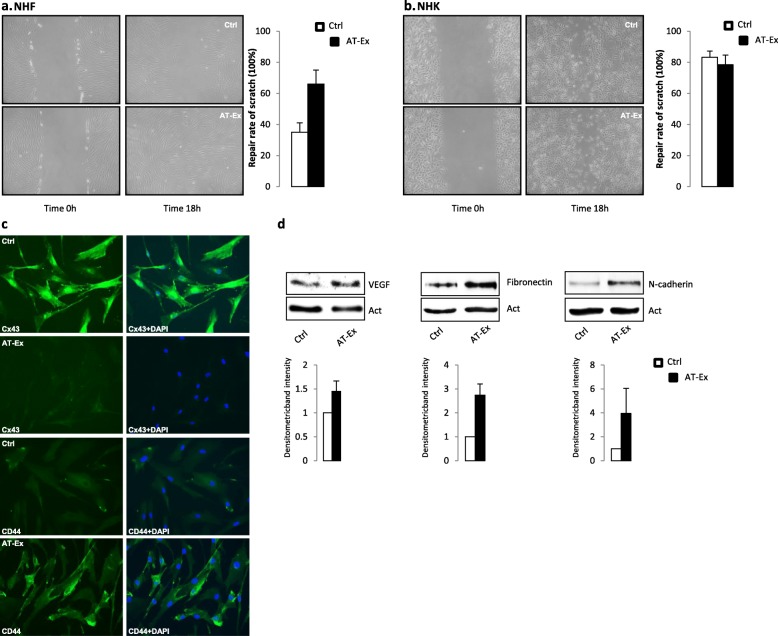
The effect of adipose tissue extracellular fraction (AT-Ex) on cell migration. Scratch assay with Normal human fibroblasts (NHF) (**a**) and normal human keratinocytes (NHK) (**b**) were performed to monitor cell motility in vitro. Both cell types were grown to confluence and starved for 24 h before the monolayer were scratched and then exposed to AT-Ex (2%) or not (control cells; Ctrl). The rate of migration was evaluated after 18 h. The extent of the wound closure is presented as the percentage by which the original scratch width has decreased. Data presented are representative of four independent experiments. **c** Following 24 h treatment, AT-Ex induces downregulation of connexin-43 (Cx-43) gap junctional protein expression in NHF and increased expression of CD44, a membrane protein associated with augmented cell motility. **d** Western blot analysis shows increments in vascular endothelial growth factor (VEGF), N-cadherin, and fibronectin proteins. Images are representative of five independent experiments. Nuclei were labeled with bisbenzidine (DAPI). Original magnification 40×. Images are representative of several independent experiments

To test the chemotactic activity of different AT-Ex, we investigated the migration ability of human fibroblasts and keratinocytes, the two major cell types involved in the wound healing process. We performed scratch assays to evaluate the wound closure after 18 h of treatment in the absence of mitogenic stimulation. In this case, to exclude the overlapping of a mitogenic effect, the cells were kept under conditions of starvation for 24 h before the start of the assay. The basal migration rate of adult keratinocytes appeared to be higher than adult fibroblasts, and the addition of AT-Ex resulted in an acceleration of the migration capacity of adult fibroblasts (Fig. 3a) whereas keratinocytes showed no difference in the regulation of cell motility in the presence or not of treatments (Fig. 3b). According to the scratch closure test results, AT-Ex treatment modulates the processes involved in wound healing, such as a reduction in the jap junctional protein connexin-43 (Cx43) and increases in N-cadherin and CD44 adhesion molecules (Fig. 3c, d) involved in fibroblast motility during tissue injury and remodeling of the extracellular matrix by stimulation of fibronectin expression [28]. Furthermore, analyses were performed to determine whether AT-Ex treatment could affect VEGF expression since neovascularization could accelerate wound closure [29]. Taken together, the observed modification of dermal fibroblasts indicated that AT-EX, administrated exogenously, could promote the remodeling phase of wound healing (Fig. 3d).

### Protective effects of AT-Ex components on chemical- and UV-damaged cells

**Fig. 4:**
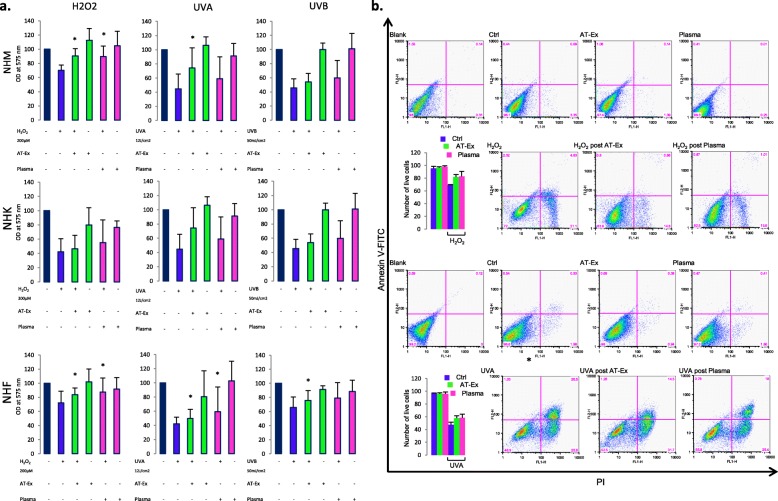
The protective effect of adipose tissue extracellular fraction (AT-Ex) on cell viability following H_2_O_2_, UVA, and UVB irradiation. **a** AT-Ex and plasma supplementation were added at the concentration of 2% (v/v) immediately after exposure to stressor. Cell viability was determined 24 h post-treatment with UV or H_2_O_2_. The data represent the mean ± SD of 12 experiments performed in duplicate; statistical significance versus untreated control (Ctrl) is reported as *p < 0.05. **b** Death and apoptosis were evaluated by FACS analysis using annexin V/iodide propidium (PI) staining. Dot plots show one representative experiment performed 24 h after H_2_O_2_ or UVA exposure. The number of surviving cells (Annexin V^–^/PI^–^) was augmented in the presence of AT-Ex and plasma. OD optical density, NHF normal human fibroblasts, NHK normal human keratinocytes, NHM normal human melanocytes

**Fig. 5:**
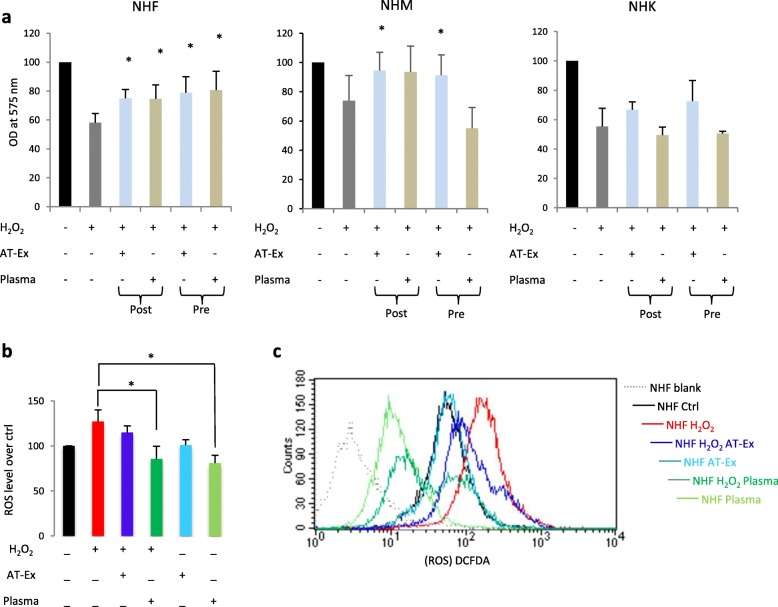
The effect of adipose tissue extracellular fraction (AT-Ex) on intracellular reactive oxygen species (ROS) clearing in normal human fibroblasts (NHF), normal human melanocytes (NHM), and normal human keratinocytes (NHK). **a** Cells were pretreated or post-treated with 2% (v/v) AT-Ex or matched plasma for 24 h and exposed to H_2_O_2_. Twenty-four hours after H_2_O_2_ treatment, cell viability was evaluated by MTT assay. Experiments were performed in triplicate; statistical significance versus untreated control (Ctrl) is reported as *p < 0.05 **b** The same experimental conditions described in (**a**) were used to quantify intracellular ROS levels using the fluorogenic probe H_2_DCF-DA by flow cytometric analysis. Histograms represent the mean ± SD of 14 (NHF) or 8 (NHK and NHM) independent experiments. **c** One representative histogram presenting the overlay of samples loaded with DCFH-DA. NHF blank = no probe loaded. OD optical density

**Fig. 6:**
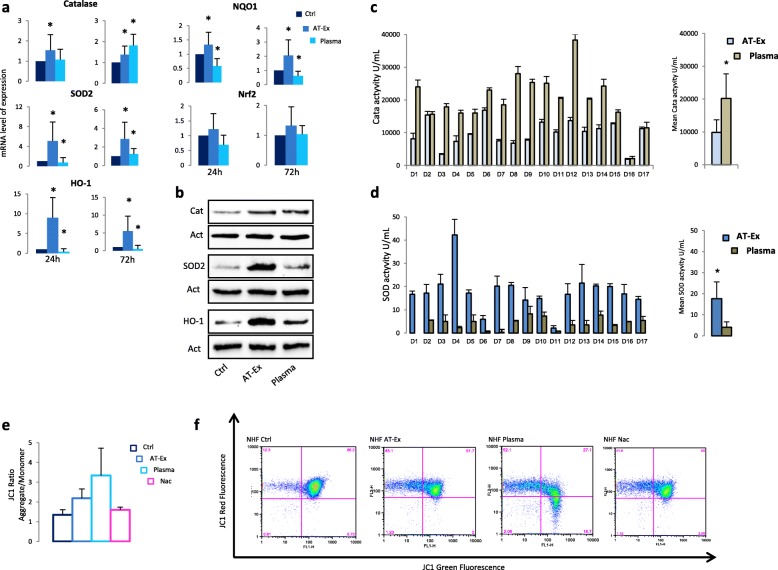
Detoxification of ROS by adipose tissue extracellular fraction (AT-Ex) and plasma supplementation in normal human fibroblasts (NHF). **a** NHF were lysed after 24 and 72 h treatment for gene expression analysis of antioxidant enzymes by semiquantitative RT-PCR. β-actin expression was used to normalized cDNA concentration for each sample set. Means and SD are from six independent experiments. Statistical significance versus untreated control (Ctrl) is reported as *p < 0.05. **b** Gene expression data were confirmed at the protein level by Western blot analysis. Catalase (Cata) (**c**) and superoxide dismutase 2 (SOD2) (**d**) activity were measured using fluorescent and colorimetric assays, respectively. Samples were diluted 1:5 prior to assaying. Experiments were performed in duplicates. Histograms report data from single donors (*n* = 17 donors) and the mean value ± SD. Statistical significance versus untreated control is reported as *p < 0.05. **e** Mitochondrial membrane potential was measured by JC-1 staining and analyzed by flow cytometry. Increase in red fluorescence (monomer)/green fluorescence (aggregate) ratio demonstrated high mitochondrial membrane potential corresponding to more energized mitochondria. Means and SD are from six independent experiments. **f** Dot plot analysis of representative experiment shows a subpopulation of cells presenting depolarized mitochondria in plasma-treated NHF. Nac N-acetylcysteine

Next, we tested the effect of AT-Ex in contracting cell damage following exposure to UVA, UVB, and H_2_O_2_ on skin and dermal cells that are physiologically exposed to these environmental stressors since both UV radiation and H_2_O_2_ enhance ROS generation, compromising mitochondrial antioxidant defense of cells and the integrity of lipids, proteins, and nucleic acids [30]. ROS also function as important physiological regulators of diverse biological parameters ranging from intracellular transduction pathways to the generation of the inflammatory response, and deregulated ROS signaling may cause or accelerate a host of human conditions including chronological aging and photo-aging. In particular, the prolonged release of excess ROS in the skin can aggravate inflammatory injury and promote chronic inflammation [31]. Moreover, increased ROS levels has been proven to be a key player during vitiligo initiation and progression [32, 33]. Depending on the cell type, we used different doses of UV and H_2_O_2_ to achieve a moderate level of cytotoxicity since in a set of preliminary experiments we demonstrated a cell type-dependent sensitivity to these stimuli (data not shown). Even though all cell populations displayed an unmistakable benefit post-treatment with AT-Ex, a significant (*p* < 0.05) protective effect was observed exclusively on NHM and on NHF, whereas on NHK we observed only a slight increase in cell vitality compared with untreated cells (Fig. 4a). In particular, AT-Ex demonstrated harmful effects in counteracting H_2_O_2_- and UVA-dependent oxidative damage and modestly impacted on direct UV-induced damage with UVB. No differences were observed in comparative analyses of AT-Ex and patient-matched plasma post-treatment. Data were also confirmed when evaluating the number of apoptotic cells by Annexin V/PI staining on fibroblasts (Fig. 4b). Using an overnight (about 18 h) pretreatment in place of post-treatment, we obtained a similar or slightly higher reduction of H_2_O_2_-induced toxicity suggesting that the mechanism of protection was not simply due to direct ROS scavenging (Fig. 5a). In contrast, pre-exposure to plasma demonstrated some effectiveness only in fibroblasts, whereas in melanocytes and keratinocytes the same pretreatment negatively influenced cell vitality. The level of ROS produced after H_2_O_2_ and UVA exposure was modestly reduced by AT-Ex treatment, whereas the addition of plasma to the culture medium strongly impacted on the level of ROS in both control and H_2_O_2_-exposed cells (Fig. 5b, c). The strength of the antioxidant capacity of plasma is also demonstrated by the fact that the decrease in intracellular ROS goes beyond the simple contrast effect of the H_2_O_2_-induced elevation, reaching values below the untreated control. Overall, the data suggested that, even if the general outcome achieved by AT-Ex and plasma supplements is similar, the biologic effects triggered with these two supplementations are not fully overlapping. Since AT-Ex treatment normalized the level of ROS production after H_2_O_2_ exposure and mildly reduced the basal amount of intracellular reactive species while plasma dramatically lowered the level of ROS, we postulated that plasma acts mainly as a direct scavenger whereas AT-Ex stimulated the physiological intracellular machinery designed for detoxification. Accordingly, after 24 h exposure to AT-Ex the expression of most of the antioxidant enzymes was upregulated at the mRNA and protein level indicating the activation of intrinsic cell defense mechanisms. To the contrary, supplementation with plasma at the same concentration modestly increased the level of antioxidant enzymes at the protein level and significantly downregulated the corresponding gene transcription with the exception of catalase (Fig. 6a, b). Downregulation of antioxidant enzyme mRNA and a simultaneous mildly increased level of the corresponding proteins could also be a consequence of the drastic reduction in ROS levels since the production of antioxidant proteins and their turnover depends on the overall balance between intracellular ROS levels and the capacity of cells to buffer these highly reactive species [34]. Accordingly, cell treatment with the free radical scavenging N-acetylcysteine (Nac) exerted a similar effect (Additional file 1: Figure S2). To investigate deeper, we measured catalase and SOD activity of lipoaspirate extracellular fraction and plasma. Data demonstrated strong and moderate catalase activity of plasma and lipoaspirate-derived fluid, respectively (Fig. 6c). To the contrary, SOD activity was significantly higher in AT-Ex compared with plasma (Fig. 6d). The mitochondrial membrane potential monitored by JC-1 fluorescent staining indicated that the JC-1 ratio (aggregate/monomer) was significantly increased by AT-Ex and plasma supplementation, indicating a higher level of energized mitochondria. However, in the case of fibroblasts grown in the presence of plasma, analysis of the dot plot clearly defined the presence of a cell population poor in JC-1 aggregates (red fluorescence indicative of energized mitochondria), presenting the same fluorescence intensity for monomers (green fluorescence indicative of depolarization) compared with control cells (Fig. 6e, f), indicating a collapse of mitochondrial transmembrane potential. The evidence of mitochondrial heterogeneity highlights the nonoverlapping outcome exerted by plasma and AT-Ex treatments.

### Anti-aging effects of AT-Ex on fibroblasts

**Fig. 7:**
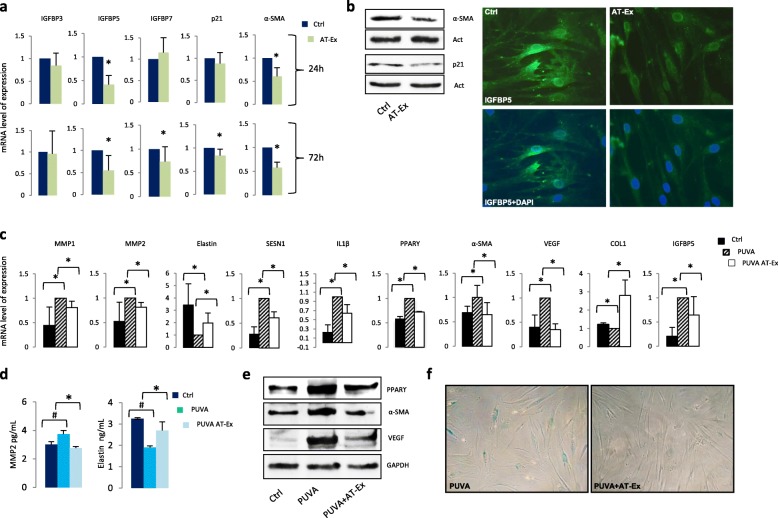
Partial reversal of cellular senescence by adipose tissue extracellular fraction (AT-Ex) supplementation in NHF. **a**, **b** After 72 h treatment, Western blotting using α-SMA and p21 antibodies and immunofluorescence analysis for IGFBP5 confirmed gene expression results. Images show representative data from three independent experiments. Similar results were obtained at 24 h (data not shown). NHF were incubated with 8-MOP for 4 h and then exposed to UVA (6 J/cm^2^) to induce premature senescence. Next, cells were grown in the presence (or not as control (Ctrl) cells) of 2% (v/v) AT-Ex (or 2% FBS for control cells) for 2 weeks. The acquisition of senescent phenotype was determined though the analysis of relevant senescence-associated markers at the mRNA (**c**) and protein level by ELISA (**d**) or Western bolt analysis (**e**). SA-β-gal assay confirmed the protective effect of AT-Ex (**f**). Quantitative data represent the mean ± SD of five independent experiments; statistical significance versus untreated control is reported as *p < 0.05

Perturbation of oxidative balance and modifications in the expression of proliferation markers in progenitor cells associated with loss of tissue repair capacity are the major phenotypes of senescent cells [35]. To examine the effects of AT-Ex on adult fibroblasts, cells were lysed after 24 and 72 h of treatment for protein and gene expression analysis. In addition to the stimulation of the antioxidant defense system, AT-Ex treatment significantly reduced the expression of functional and structural proteins associated with the fibroblast senescent phenotype, such as IGFBP5 and 7, αSMA, and p21 (Fig. 7a, b). We additionally investigated the ability of AT-Ex to counteract stress-induced premature senescence (SIPS) achieved by a single exposure of dermal fibroblasts to UVA and 8-methoxypsoralen (PUVA). As we previously reported, PUVA treatment activates a senescence-like phenotype associated with long-term growth arrest, flattened morphology, and increased synthesis of MMPs [36]. AT-Ex administration in PUVA-treated NHF significantly prevented the biological modification typical of the senescence phenotype [37], such as upregulation of IGFPB5, MMP1, MMP2, and α-SMA expression and lower elastin and collagen production (Fig. 7c). Further evidence of the AT-Ex antisenescence effect was the abrogation of the proinflammatory senescent-associated secretory phenotype (SASP), such as the cytokines interleukin (IL)-1β and VEGF and the absence of lysosomal β-galactosidase activity (Fig. 7c, d, f). Additionally, the expression of peroxisome proliferator-activated receptor γ (PPARγ), a nuclear receptor that plays a relevant role in the accelerated aging process [38, 39], was reduced by AT-Ex in PUVA-treated cells (Fig. 7c, d). Sestrin-1 (SESN1), a member of a family of proteins that also includes SESN2 and SESN3 [40] that confer resistance to oxidative stress, was reasonably induced compared with control PUVA-treated cells, demonstrating a moderate perturbation of intracellular redox equilibrium. Altogether, these data indicate that AT-Ex supplementation mitigated the PUVA effects, ameliorating all the typical features of photo-aging.

### Immunoenzymatic analysis of adipose tissue-associated extracellular fluid revealed a specific profile rich in growth factors

Recently published studies have demonstrated that ADSCs produce complementary wound healing trophic factors including insulin-like growth factor (IGF), bFGF, HGF, VEGF, KGF, and Wnt10b [41, 42]. A number of these growth factors and other important mediators of skin regeneration were investigated using immunoenzymatic quantification, as shown in Table 1. Most of the growth factors quantified resulted higher levels, or were exclusively present, in AT-Ex in comparison with plasma, indicating that even if liposuction is an invasive procedure that causes broken capillaries, active molecules derive mainly from adipose tissue. In particular, bFGF, nerve growth factor (NGF), and VEGF were specifically detected in AT-Ex samples. Additionally, we evaluated the presence of α-MSH and SCF, two important factors in the regulation of melanocyte differentiation and survival [43, 44]. We also demonstrated that two modulators of the Wnt/β-catenin pathway, Wnt3a and Wnt10b, implicated in replicative senescence regulation [45] and in wound healing processes [46, 47], respectively, are abundantly present in lipoaspirates. Overall, our data demonstrated a specific signature of adipose tissue secretome that does not overlap with circulating factors.

**Table 1:** Determinations by ELISA for each factor analyzed, with the range of values observed among positive samples, the number of positive samples in each group, and the detection limit of assays

ELISA targets	AT-Ex			Plasma			Detection limit
	No. of positive samples	Mean ± SD	Range	No. of positive samples	Mean ± SD	Range	
bFGF	20/20	4430.7 ± 3102.9	1015.5–11476.0	0/20	ND	ND	50 pg/ml
EGF	20/20	35.3 ± 21.0	15.5–63.1	20/20	562.5 ± 186.1	160.8–772.6	26 pg/ml
Epo	20/20	5.24 ± 1.87	2.26–8.6	20/20	7.83 ± 4.9	3.0–16.9	0.17 mlU/ml
GM-CSF	17/20	2.17 ± 1.38*	01.6–4.4*	20/20	10.2 ± 11.0	2.0–26.6	0.6 pg/ml
KGF	17/20	486.3 ± 279.5*	176.4–1052.0*	8/20	602.4 ± 612.6*	150.0–1299.6	4.0 pg/ml
SCF	20/20	5.79 ± 2.82	1.6–10.4	0/20	ND	ND	0.45 ng/ml
NGF	20/20	95.2 ± 9.04	84.0–112.0	0/20	ND	ND	19.5 pg/ml
VEGF-A	20/20	2274.7 ± 964.7	315.8–2885.0	17/20	337.8 ± 243.3*	0–775.3*	7.9 pg/ml
α-MSH	20/20	2.16 ± 1.13	0.46–3.91	14/20	0.067 ± 0.043*	0.029–0.146	0.039 ng/ml
Wnt3a	20/20	21.6 ± 2.4	9.82–28.0	4/20	0.50 ± 0.45*	0.08 ± 1.25	0.054 ng/ml
Wnt10b	19/20	4.0 ± 1.26*	0.24–4.25	0/20	ND	ND	0.058 ng/ml

*bFGF* basic fibroblast growth factor, *EGF* epidermal growth factor, *ELISA* enzyme-linked immunosorbent assay, *Epo* erythropoietin, *GM-CSF* granulocyte/macrophage colony-stimulating growth factor, *KGF* keratinocyte growth factor, *MSH* melanocyte stimulating hormone, *ND* not determined, *NGF* nerve growth factor, *SCF* stem cell factor, *VEGF* vascular endothelial growth factor, *Wnt* wingless type

*mean values were calculated only with positive samples whereas range of concentration include negative sample (negative=0)

### Technical considerations for clinical application

**Fig. 8:**
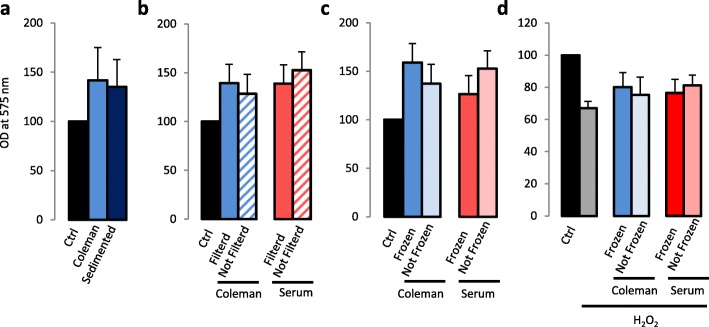
Technical aspects of AT-Ex preparation. **a** Analysis of the effect of centrifugation versus a sedimentation step on NHF proliferation during the preparation of AT-Ex and plasma. **b** Evaluation of the impact of the filtration step during AT-Ex and plasma sample preparation on NHF fibroblast proliferation. **c** Evaluation of AT-Ex and plasma banking at low temperature (−80 °C). The biological effect of frozen versus not frozen AT-Ex and plasma were investigated measuring the impact on NHF proliferation or on cell viability following H_2_O_2_ exposure. Histograms represent the mean ± SD of six independent experiments; statistical significance versus untreated control (Ctrl) is reported as *p < 0.05. OD optical density

Lipoaspirate-associated fluids are produced intraoperatively as waste material since most surgical protocols for fat grafting recommend a condensation of ADSCs. The relevance of graft tissue condensation is related to graft material retention and to the limitation of the injected volume since an injection of excessive volume leads to severe ischemia and fat necrosis [2]. ADSCs can be condensed while excluding the liquid fraction by a decantation (gravity sedimentation) or by a centrifugation step. Comparative analysis of the biological activity of AT-Ex prepared by the method of Coleman and Katzel [22], the most popular centrifugation protocols for clinical purposes, or by a simple sedimentation step did not show any significant differences (Fig. 8a). We also tested the effect of the filtration step that we used to remove solid particles and to guarantee the absence of residual cells. In this case, results showed increased biologic effects, as measured in terms of cell proliferation, following a passage through a 0.22-μm filter system suggesting that larger suspended particles could be broken, releasing active biomolecules (Fig. 8b). Moreover, we compared fresh AT-Ex preparations to matched frozen and thawed samples and we detected no decrease in terms of stimulation of cell proliferation and protective capacity (Fig. 8c, d). In contrast, similar to filtration, we observed a slight increase in the proliferative capacity. Storage time also should not influence AT-Ex biological activity since long-term cryopreservation (2 years) did not reveal significant difference with respect to short-term cryopreservation (1 h) (data not shown).

## Discussion

Since the discovery of ADSCs in human adipose tissue nearly 15 years ago, significant advances have been made in progressing this promising cell therapy tool from the laboratory bench to the bedside. Recently, focus has shifted to the immune modulatory and paracrine effects of transplanted ADSCs, with growing interest in the ADSC secretome as a source of clinical effect. The use of cell-free therapies such as the ADSC secretome in regenerative medicine provides key conveniences over stem-cell based applications—administration of the secretome resolves several safety problems potentially associated with the transplantation of living and proliferative cell populations including tumorigenicity, transmission of infections, and immune compatibility. In addition, storage can be carried out without extensive manipulation and potentially toxic cryopreservative agents for a long period without loss of product potency. The possibility for performing repeated therapeutic intervention is of particular interest for skin diseases due to the easy access to damage tissue. In this study, we show for the first time that the physiological secretome of adipose tissue in the form of a liquid extracellular fraction of lipoaspirate is an efficient agent in an in-vitro model for skin regeneration. AT-Ex positively impacts on cell proliferation, cell migration, and cell senescence in human skin cells, three critical parameters associated with lesion repair and tissue regeneration. Comparative analysis with patient-matched plasma demonstrated that even if plasma shares some beneficial effects with AT-Ex, plasma diminished the proliferation of keratinocytes and showed no effect on melanocyte growth. Moreover, in fibroblasts supplemented with plasma, independent of the changes in the protein levels of the antioxidants, a decrease at the transcription level was observed. Plasma reduced the mRNA levels of SOD2, HO-1, NQO1, and Nrf2 by approximately 26%, 60%, 43%, and 30% respectively, compared with the untreated fibroblasts. In contrast, and in line with previous studies [48, 49], the peroxisomal antioxidant enzyme catalase was regulated mainly at the protein level. The strong reduction in antioxidant mRNAs presumably exposes cells to dramatic consequences in case of sudden external injury, as demonstrated by the failure of plasma pretreatment in NHK and NHM. Thus, the extrinsic reduction of intracellular ROS achieved by plasma treatment seems to downregulate autonomous cell defense whereas the intrinsic stimulation of the antioxidant system obtained with AT-Ex exposure led to a more physiological control of the ROS balance capable of counteracting injury. ROS regulate several biological processes and the loss of antioxidant equilibrium is implicated in various pathological conditions. Increased levels of oxygen species disrupt the mechanisms of wound healing, and supplementation with antioxidants has been proposed for nonhealing wound therapy. Thus, the beneficial enhancement of antioxidant intracellular defense exerted by AT-Ex could provide a relevant improvement in indolent nonhealing wounds. On the other hand, increased ROS level has been proven to be a key player during initiation and progression of vitiligo, a common skin condition characterized by melanocyte disappearance in the lesional area [32, 33]. Among the possible therapeutic approaches, an autologous noncultured epidermal cell suspension is used as an effective cell-based therapy. As an innovative strategy, releasing a melanocyte suspension into AT-Ex before transplanting them into depigmented recipient skin could ameliorate cell engraftment and improve the surgical management of vitiligo. The stimulation of the endogenous antioxidant network is probably the mechanism mediating the protection to the propagation of PUVA-SIPS since the resultant robust induction of all the senescence-associated markers was mitigated by AT-Ex supplementation. Consistently, SESN1, a protein responsible for resistance to oxidative stress [50], was slightly induced in AT-Ex-treated cells, indicating attenuation of the detrimental consequences of chronic exposure to subtoxic oxidative stress.

Previous studies demonstrated that both ADSCs and adipocytes secrete a multitude of growth factors, hormone-like proteins, and adipokines [11, 12, 15], and a comparative analysis of ADSC-, adipocyte-, and adipose tissue-conditioned medium showed comparable potency in wound healing suggesting the use of whole adipose tissue rather than isolating ADSCs or adipocytes [16]. By demonstrating the potency of AT-Ex, and as it can be isolated by a rapid intraoperative centrifugation and filtration step (about 15 min), we propose the use of adipose secretome for clinical application in the treatment of acute and chronic wounds, burns, and ulcers, avoiding complicated and time-consuming procedures. Moreover, since AT-Ex demonstrated harmful effects in counteracting oxidative damage, we also propose a possible therapeutic application to correct the impaired intracellular redox balance of vitiligo cells since the alteration of redox equilibrium contributes in synergy with autoimmunity to the loss of functional melanocytes and melanin from epidermis [51–53]. Ongoing experiments have confirmed the results on vitiligo melanocytes isolated from nonlesional areas (unpublished data). In addition to the cell-free approach, an intriguing clinical application is the improvement of current surgical options for vitiligo treatment combining AT-Ex with autologous cultured melanocytes, noncultured epidermal cell suspension, or with pluripotent ADSCs capable of differentiating into the melanocyte lineage [23, 54]. We recently successfully used fat minigraft in combination with autologous noncultured epidermal cell suspension transplantation to correct skin scarring occurring following skin cancer resection [55]. In this case, we used the patient’s plasma as a natural carrier for the cells. This procedure, which showed better results with respect to an autologous full-thickness skin graft, could be improved in the future by the replacement of plasma with AT-Ex. More generally, considering all current clinical applications in regenerative medicine, AT-Ex could be used as an adjuvant therapy in combination with autologous fat tissue or purified ADSCs when temporally induced proliferation of grafted (and resident) stem cells in the damaged tissue is desirable. In fact, the modulation of a hostile environment during illness could show a beneficial effect for stem cell persistence. For this purpose, in addition to proliferation, we also looked at the differentiation potential of AT-Ex-treated ADSCs and we did not observe any significant modification in adipogenic and osteogenic differentiation (data not shown). In agreement with this idea, the use of platelet-rich plasma (PRP) in combination with mesenchymal stem cells has been suggested for skin wound healing [56, 57] since the high content of growth factors is capable of inducing ADSC and fibroblast proliferation [58]. However, lipoaspirates containing the naive microenvironment of adipose stem cells could represent a more physiological context to support stem cell graft. This hypothesis is supported by the demonstration that an array of interesting factors, such as Wnts, are absent or barely detected in plasma.

Increasing evidence indicates that ADSCs produce massive amounts of exosomes in comparison with other cells, and that many of the regenerative properties previously credited to stem cells are shown to be mediated through secreted exosomes [59]. Moreover, treatment with mesenchymal stem cell-derived exosomes and microvesicles improves at least one clinically relevant parameter associated with organ functionality including cutaneous regeneration and wound healing [14]. In our study, the slight but reproducible increase in biological activity of AT-Ex following a freeze and thaw cycle or a filtration step suggests that active factors could be, at least in part, encapsulated in extracellular vesicles secreted by adipocytes, ADSCs, or other adipose tissue-resident cells. Even if future studies are necessary to investigate these aspects, it appears evident that clinically applicable cryopreservation and banking (under cGMP conditions) of AT-Ex offers unique opportunities to advance the potential uses and widespread implementation of these treatments.

AT-Ex obtained from lipoaspirate contains an array of trophic factors comprising VEGF, KGF, Wnt3a, and α-MSH at higher concentrations and hematopoietic factors (Epo and GM-CSF) at concentrations similar to plasma. Among the growth factors investigated, only EGF was more abundant in blood samples. More interestingly, bFGF, NGF, SCF, and Wnt10b have been detected exclusively in the lipoaspirate liquid fraction. The presence of Wnts in the lipoaspirate is of particular interest since the activation of Wnt/β-catenin contributes to the wound healing process [47], delays the senescence process of mesenchymal stem cells [40], and protects from neurodegeneration [60, 61]. Our data demonstrated that the vast array of bioactive factors contained in lipoaspirate could support skin repair by two synergic mechanisms: directly, by modulation of relevant cell parameters such as viability, proliferation, and migration of the damaged tissue, and by modulation of the secretory activity of dermal fibroblasts. The plasticity of AT-Ex treatment is also demonstrated by the different effects observed for VEGF expression in normal fibroblasts and in stress-induced senescent fibroblasts. In the first case, AT-Ex slightly increased VEGF expression, as is desirable to support tissue wounds; instead, in photodamaged senescent cells expressing high level of VEGF, AT-Ex treatment rebalances VEGF production. Similar results were observed with metalloproteinases (data not shown), important modulators of extracellular matrix remodeling and of the re-epithelialization process.

## Conclusions

In conclusion, this study provides preclinical evidence that the liquid material of lipoaspirates, currently discarded as a waste by-product, is rich in biologic elements with protective and reparative properties conferring cellular benefits. We propose that the extracellular fraction of lipoaspirates could be used as an innovative therapy for specific applications requiring minimal or no soft tissue augmentation but functional or structural tissue repair such as skin diseases (acute and chronic wounds, burns, ulcers, and vitiligo). More generally, by providing evidence that adipose-derived secretome also stimulates ADSCs, we suggest a possible adjuvant therapy in combination with autologous fat tissue or purified ADSCs when temporally induced proliferation of grafted (and resident) stem cells in the damaged tissue is desirable. This work is an important stepping stone towards the development of personalized treatments in regenerative medicine based on autologous graft of native adipose tissue secretome without in-vitro manipulation.

## Additional file

Additional file 1: **Figure S1.** Cell proliferation of AT-Ex-treated cells. (a) NHF, (b) NHK, (c) NHM, and (d) ADSCs were harvested by incubation in 0.5% trypsin, 0.2% ethylenediaminetetraacetic acid (EDTA) at 37 °C. Cell viability was measured by Trypan blue exclusion assay. Histograms represent the number of viable cells after 72 h exposure to AT-Ex at different concentrations (1%, 2%, 5%, and 10% v/v in starved medium). Experiments were performed three times. Graphs represent the mean ± SD of three independent experiments; statistical significance versus untreated control is reported as *p < 0.05. **Figure S2.** (a) Phase-contrast microscopic analysis of NHK treated with AT-Ex or plasma (2%) for 72 h evidenced by marked morphological differences and a more compact distribution in the presence of plasma. (b) Immunofluorescence analysis of E-cadherin expression. Plasma supplementation impacted on the localization of E-cadherin increasing at the placement at the cell-cell contact. Nuclei were labeled with bisbenzidine (DAPI). Original magnification 40×. Images are representative of several independent experiments. **Figure S3.** N-acetylcysteine (Nac) reduced antioxidant enzymes at the mRNA level. The mRNA levels of catalase, NQO1, Nrf2, SOD2, and HO-1 were measured by semiquantitative RT-PCR after 24 h incubation with Nac, AT-Ex, or plasma. Untreated control cells were used as a reference. β-actin expression was used to normalized cDNA concentration for each sample set. Graphs represent the mean ± SD of three independent experiments; statistical significance versus untreated control is reported as *p < 0.05. **Table S1.** Primers for RT-PCR. (PPTX 5611 kb)

## Abbreviations

ADSC: Adipose-derived stem cell
AT-Ex: Adipose tissue extracellular fraction
bFGF: Basic fibroblast growth factor
Cx43: Connexin-43
Epo: Erythropoietin
HO-1: Heme oxygenase 1
IGFBP: Insulin-like growth factor binding protein
KGF: Keratinocyte growth factor
MMP: Matrix metalloproteinase
MSH: Melanocyte stimulating hormone
Nac: N-acetylcysteine
NGF: Nerve growth factor
NHF: Normal human fibroblasts
NHK: Normal human keratinocytes
NHM: Normal human melanocytes
NQO1: NAD(P)H quinone dehydrogenase 1
Nrf2: Nuclear factor (erythroid-derived 2)-like 2
PPARγ: Peroxisome proliferator-activated receptor γ
ROS: Reactive oxygen species
SCF: Stem cell factor
SIPS: Stress-induced premature senescence
SMA: Smooth muscle actin
SOD: Superoxide dismutase
SVF: Stromal vascular fraction
VEGF: Vascular endothelial growth factor
Wnts: Wingless type
