# Supplementary material for: Adipose tissue-derived extracellular fraction characterization: biological and clinical considerations in regenerative medicine
Source: Stem Cell Res Ther. 2018 Aug 9;9:207. doi: 10.1186/s13287-018-0956-4 (PMC6085647; doi:10.1186/s13287-018-0956-4)
Supplement: Supplementary file 1 — Figure S1. Cell proliferation of AT-Ex-treated cells. (a) NHF, (b) NHK, (c) NHM, and (d) ADSCs were harvested by incubation in 0.5% trypsin, 0.2% ethylenediaminetetraacetic acid (EDTA) at 37 °C. Cell viability was measured by Trypan blue exclusion assay. Histograms represent the number of viable cells after 72 h exposure to AT-Ex at different concentrations (1%, 2%, 5%, and 10% v/v in starved medium). Experiments were performed three times. Graphs represent the mean ± SD of three independent experiments; statistical significance versus untreated control is reported as *p < 0.05. Figure S2. (a) Phase-contrast microscopic analysis of NHK treated with AT-Ex or plasma (2%) for 72 h evidenced by marked morphological differences and a more compact distribution in the presence of plasma. (b) Immunofluorescence analysis of E-cadherin expression. Plasma supplementation impacted on the localization of E-cadherin increasing at the placement at the cell-cell contact. Nuclei were labeled with bisbenzidine (DAPI). Original magnification 40×. Images are representative of several independent experiments. Figure S3. N-acetylcysteine (Nac) reduced antioxidant enzymes at the mRNA level. The mRNA levels of catalase, NQO1, Nrf2, SOD2, and HO-1 were measured by semiquantitative RT-PCR after 24 h incubation with Nac, AT-Ex, or plasma. Untreated control cells were used as a reference. β-actin expression was used to normalized cDNA concentration for each sample set. Graphs represent the mean ± SD of three independent experiments; statistical significance versus untreated control is reported as *p < 0.05. Table S1. Primers for RT-PCR. (PPTX 5611 kb) [file 13287_2018_956_MOESM1_ESM.pptx]

## Slide 1
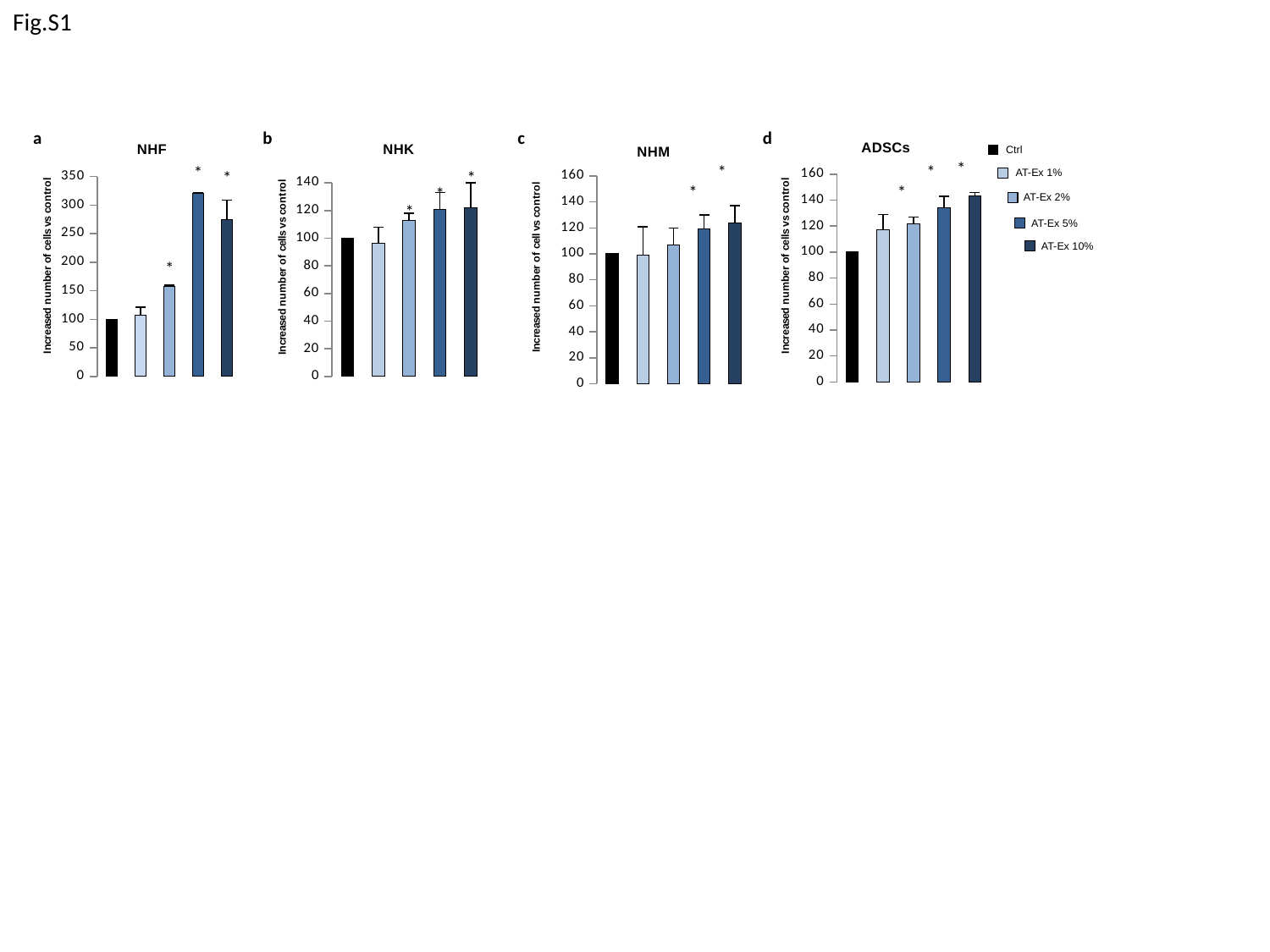

Fig.S1
a
b
c
d
### Chart: ADSCs
| Category | |
|---|---|
### Chart: NHF
| Category | |
|---|---|
### Chart: NHK
| Category | |
|---|---|
### Chart: NHM
| Category | |
|---|---|Ctrl
*
*
*
*
*
*
AT-Ex 1%
*
*
*
AT-Ex 2%
*
AT-Ex 5%
AT-Ex 10%
*

## Slide 2
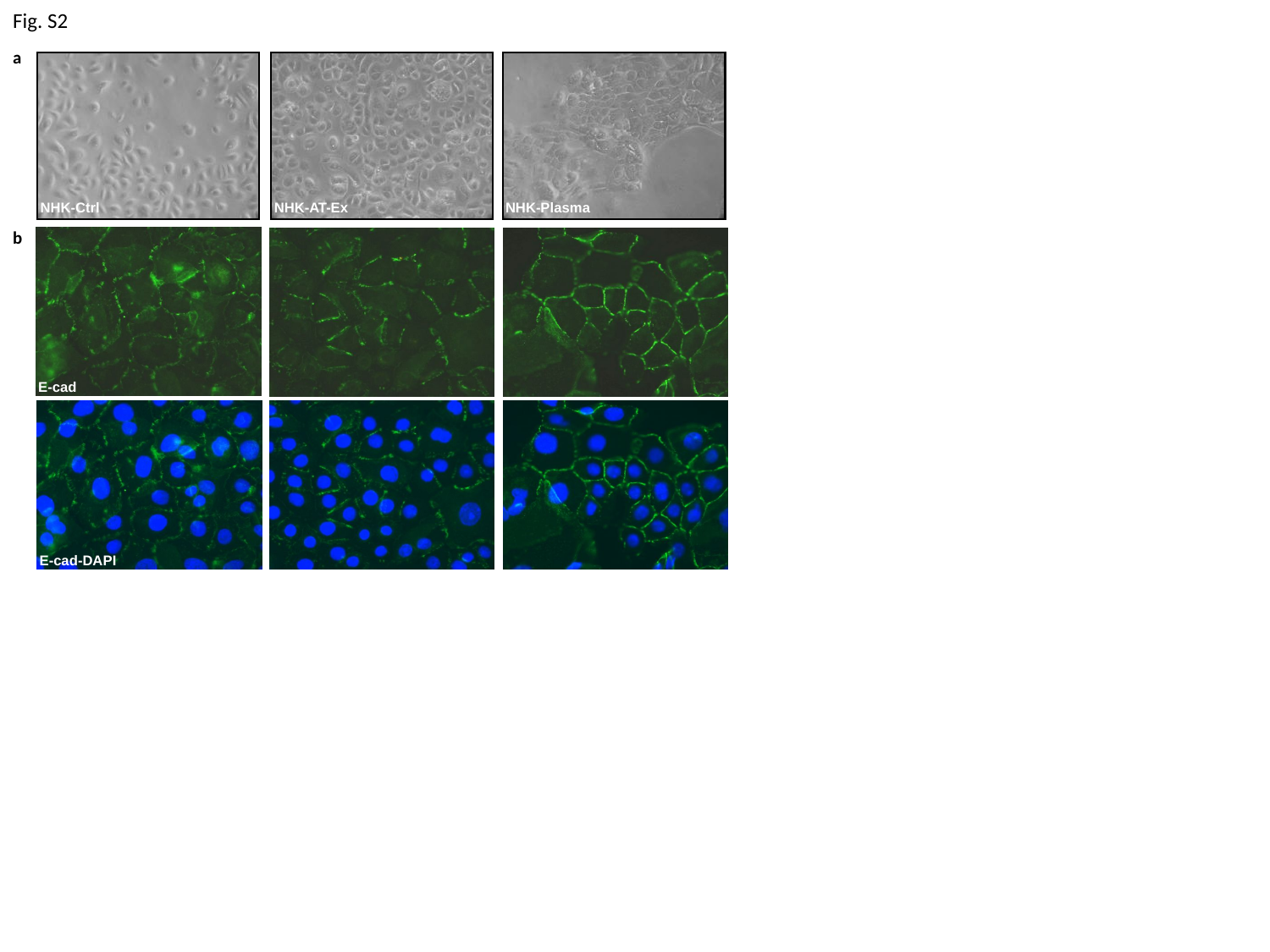

Fig. S2
a
NHK-Ctrl
NHK-AT-Ex
NHK-Plasma
b
E-cad
E-cad-DAPI

## Slide 3
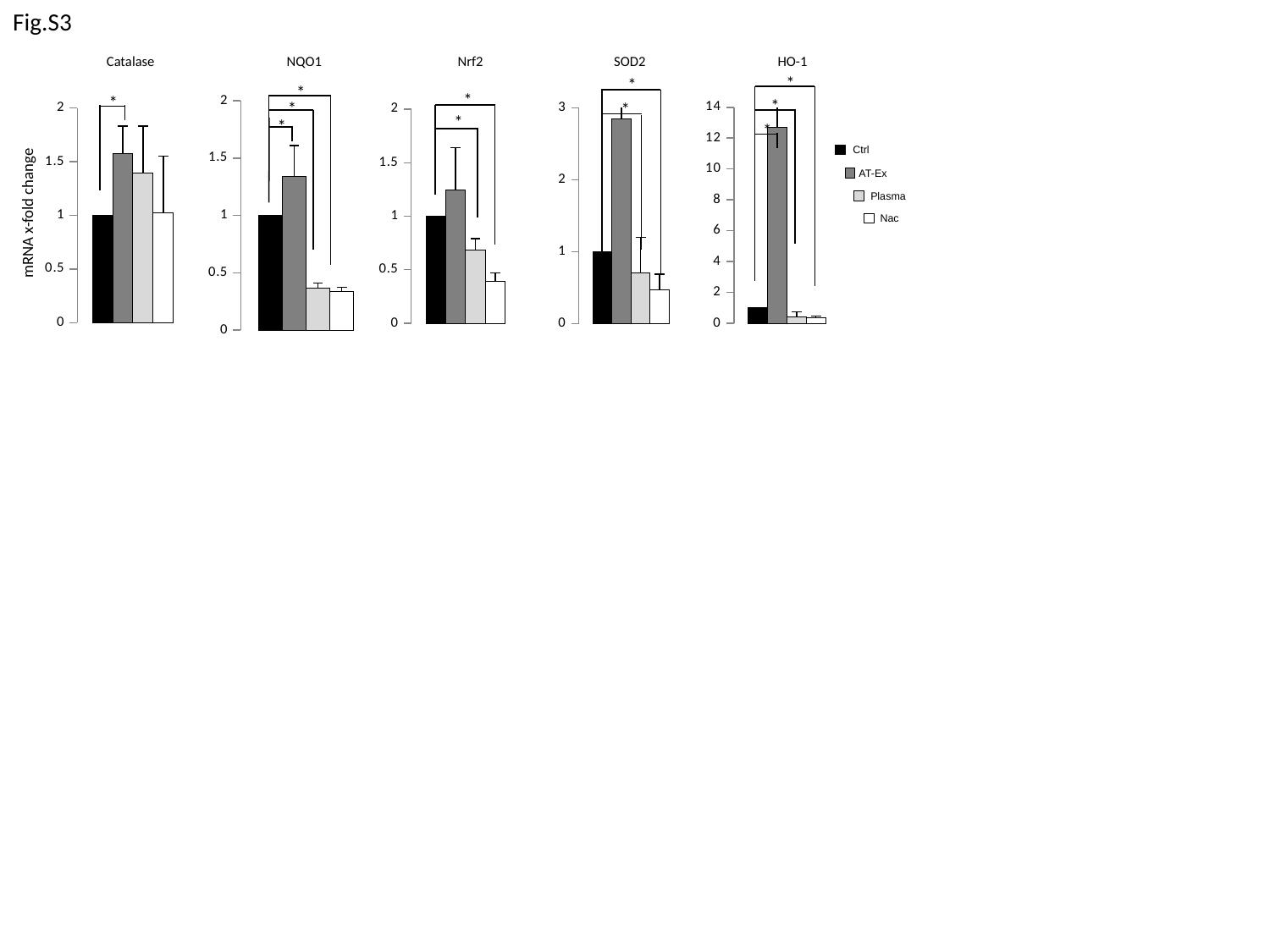

Fig.S3
Catalase
NQO1
Nrf2
SOD2
HO-1
*
*
*
### Chart
| Category | | | | |
|---|---|---|---|---|
### Chart
| Category | | | | |
|---|---|---|---|---|*
*
### Chart
| Category | | | | |
|---|---|---|---|---|*
### Chart
| Category | | | | |
|---|---|---|---|---|*
*
### Chart
| Category | | | | |
|---|---|---|---|---|*
*
*
Ctrl
AT-Ex
Plasma
mRNA x-fold change
Nac

## Slide 4
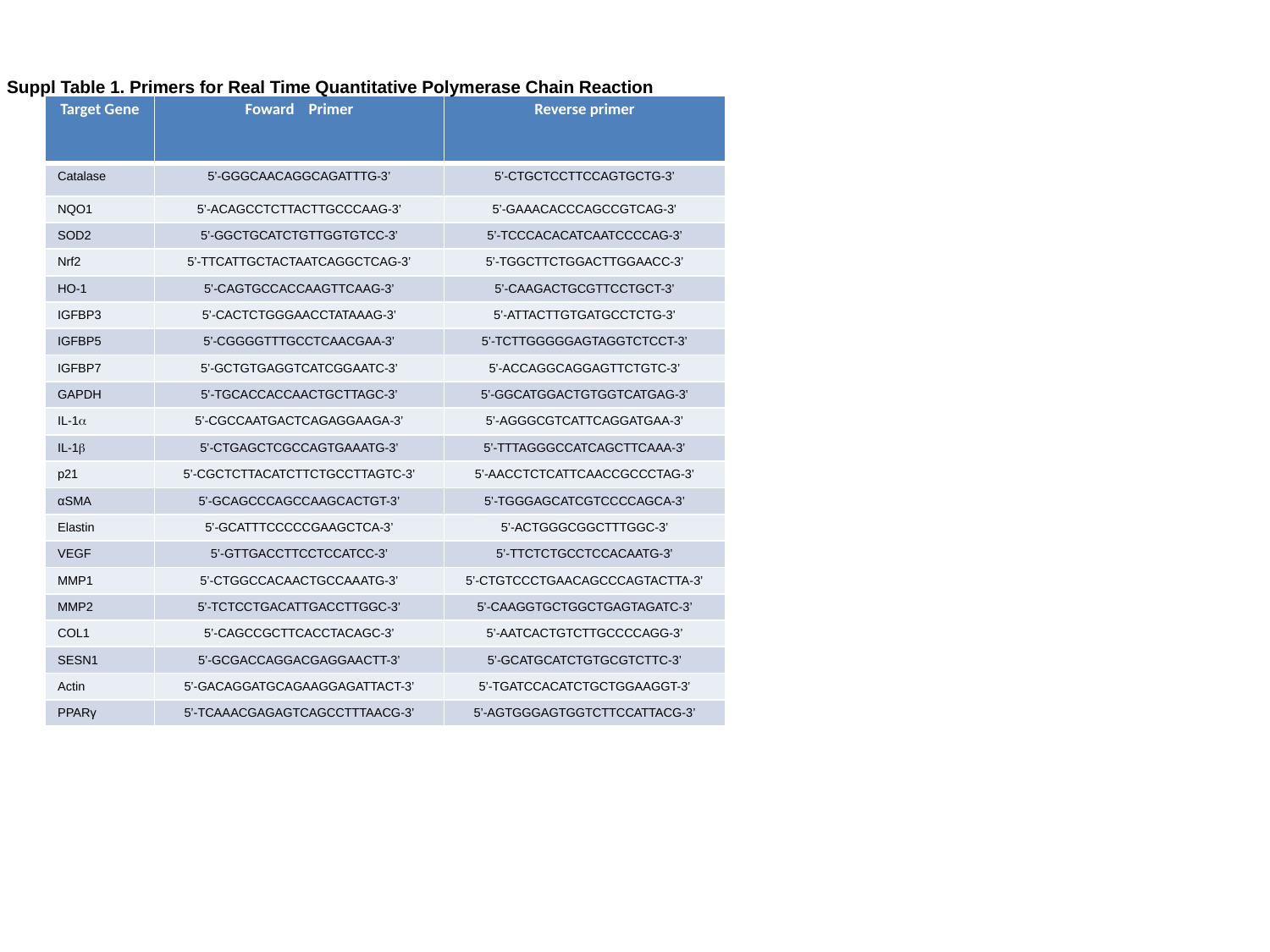

Suppl Table 1. Primers for Real Time Quantitative Polymerase Chain Reaction
| Target Gene | Foward Primer | Reverse primer |
| --- | --- | --- |
| Catalase | 5’-GGGCAACAGGCAGATTTG-3’ | 5’-CTGCTCCTTCCAGTGCTG-3’ |
| NQO1 | 5’-ACAGCCTCTTACTTGCCCAAG-3’ | 5’-GAAACACCCAGCCGTCAG-3’ |
| SOD2 | 5’-GGCTGCATCTGTTGGTGTCC-3’ | 5’-TCCCACACATCAATCCCCAG-3’ |
| Nrf2 | 5’-TTCATTGCTACTAATCAGGCTCAG-3’ | 5’-TGGCTTCTGGACTTGGAACC-3’ |
| HO-1 | 5’-CAGTGCCACCAAGTTCAAG-3’ | 5’-CAAGACTGCGTTCCTGCT-3’ |
| IGFBP3 | 5’-CACTCTGGGAACCTATAAAG-3’ | 5’-ATTACTTGTGATGCCTCTG-3’ |
| IGFBP5 | 5’-CGGGGTTTGCCTCAACGAA-3’ | 5’-TCTTGGGGGAGTAGGTCTCCT-3’ |
| IGFBP7 | 5’-GCTGTGAGGTCATCGGAATC-3’ | 5’-ACCAGGCAGGAGTTCTGTC-3’ |
| GAPDH | 5’-TGCACCACCAACTGCTTAGC-3’ | 5’-GGCATGGACTGTGGTCATGAG-3’ |
| IL-1α | 5’-CGCCAATGACTCAGAGGAAGA-3’ | 5’-AGGGCGTCATTCAGGATGAA-3’ |
| IL-1β | 5’-CTGAGCTCGCCAGTGAAATG-3’ | 5’-TTTAGGGCCATCAGCTTCAAA-3’ |
| p21 | 5’-CGCTCTTACATCTTCTGCCTTAGTC-3’ | 5’-AACCTCTCATTCAACCGCCCTAG-3’ |
| αSMA | 5’-GCAGCCCAGCCAAGCACTGT-3’ | 5’-TGGGAGCATCGTCCCCAGCA-3’ |
| Elastin | 5’-GCATTTCCCCCGAAGCTCA-3’ | 5’-ACTGGGCGGCTTTGGC-3’ |
| VEGF | 5’-GTTGACCTTCCTCCATCC-3’ | 5’-TTCTCTGCCTCCACAATG-3’ |
| MMP1 | 5’-CTGGCCACAACTGCCAAATG-3’ | 5’-CTGTCCCTGAACAGCCCAGTACTTA-3’ |
| MMP2 | 5’-TCTCCTGACATTGACCTTGGC-3’ | 5’-CAAGGTGCTGGCTGAGTAGATC-3’ |
| COL1 | 5’-CAGCCGCTTCACCTACAGC-3’ | 5’-AATCACTGTCTTGCCCCAGG-3’ |
| SESN1 | 5’-GCGACCAGGACGAGGAACTT-3’ | 5’-GCATGCATCTGTGCGTCTTC-3’ |
| Actin | 5’-GACAGGATGCAGAAGGAGATTACT-3’ | 5’-TGATCCACATCTGCTGGAAGGT-3’ |
| PPARγ | 5’-TCAAACGAGAGTCAGCCTTTAACG-3’ | 5’-AGTGGGAGTGGTCTTCCATTACG-3’ |
